# Potential of four corn varieties at different harvest stages for silage production in Malaysia

**DOI:** 10.5713/ajas.18.0175

**Published:** 2018-12-21

**Authors:** Muhamad Hazim Nazli, Ridzwan Abdul Halim, Amin Mahir Abdullah, Ghazali Hussin, Anjas Asmara Samsudin

**Affiliations:** 1 Department of Crop Science, Faculty of Agriculture, Universiti Putra Malaysia, Serdang, Selangor 43400, Malaysia; 2 Department of Agribusiness and Bioresource Economics, Faculty of Agriculture, Universiti Putra Malaysia, Serdang, Selangor 43400, Malaysia; 3 Livestock Science Research Centre, Malaysian Agriculture Research and Development Institute (MARDI), Serdang, Selangor 43400, Malaysia; 4 Department of Animal Science, Faculty of Agriculture, Universiti Putra Malaysia, Serdang, Selangor 43400, Malaysia

**Keywords:** Corn Variety, Harvest Stage, Corn Silage, Tropical, Malaysia

## Abstract

**Objective:**

Apart from various climatic differences, corn harvest stage and varieties are two major factors that can influence the yield and quality of corn silage in the tropics. A study was conducted to determine the optimum harvest stage of four corn varieties for silage production in Malaysia.

**Methods:**

Corn was harvested at four growth stages; silking, milk, dough, and dent stages from four varieties; Sweet Corn hybrid 926, Suwan, breeding test line (BTL) 1 and BTL 2. Using a split plot design, the treatments were then analysed based on the plant growth performance, yield, nutritive and feeding values followed by a financial feasibility study for potential commercialization.

**Results:**

Significant differences and interactions were detected across the parameters suggesting varying responses among the varieties towards the harvest stages. Sweet Corn was best harvested early in the dough stage due to high dry matter (DM) yield, digestible nutrient, and energy content with low fibre portion. Suwan was recommended to be harvested at the dent stage when it gave the highest DM yield with optimum digestible nutrient and energy content with low acid detergent fibre. BTL 1 and BTL 2 varieties can either be harvested at dough or dent stages as the crude protein, crude fibre, DM yield, DM content, digestible nutrient and energy were not significantly different at either stage. Further financial analysis showed that only Sweet Corn production was not financially feasible while Suwan had the best financial appraisal values among the grain varieties.

**Conclusion:**

In conclusion, only the grain varieties tested had the potential for silage making according to their optimum harvest stage but Suwan is highly recommended for commercialization as it was the most profitable.

## INTRODUCTION

Lack of good quality feed resources is one of the major factor suppressing Malaysia’s beef industry growth [1]. As a result, the national beef self-sufficiency level has dropped since 2010 and in 2016, the beef production was less than 25% of the national requirement [2]. Farmers must use more concentrates than roughages due to lack of pasture area available for grazing [3]. Feed is the major cost of beef production and the current feeding system relies heavily on expensive feed with seasonal usage of fodder grasses while corn silage is very rarely utilised. Currently, no formal production data on corn silage production is available in Malaysia due to very low production [4]. Compared to the conventional feeding system, corn silage is a competitive alternative as it has many advantages for use as ruminant feed. Locally grown corn can provide consistent high-quality feed material which can be stored over a long period as silage. Compared to labour intensive cut and carry fodder grass, corn planting can be mechanised to produce large quantities of feed and the quality can be easily controlled on farm. Apart from that, the usage of expensive concentrates can also be reduced through the concentrates sparing effect of corn silage [5].

Corn harvest stage is crucial as it influences the quality and quantity of the silage material. Efficient utilization of the silage by animals largely depends on the maturity stage at which the crop is harvested. The growth stage has a major influence on the silage digestibility and the amount consumed by livestock. Corn is the only forage that does not decrease in quality as it matures, because the grain development on the cob compensates the high fibre levels in the leaf and stem. The stages also cause variability in the DM content making the nutritional values different [6]. Various studies have shown that the best growth stage to harvest the crop is usually a compromise between the yield and quality of forage. When making silage, the biggest concern is the plant nutritional values effect on the fermentation quality. Poor fermentation can lead to high DM loss, low aerobic stability and reduction in the latter silage quality for livestock feeding. Apart from that, the varying genetic background of different corn varieties can also significantly affect the plant yield and quality. Schroeder [7] mentioned that corn hybrid selection can influence the corn silage in several ways; the yield harvested, grain content at harvest time and the digestibility. Plant breeders tend to do selection in breeding via the grain yield and most of the breeders suggested that the best grain varieties are also the best forage varieties [8]. Ferraretto et al [9] found that leafy corn hybrid appeared to have a significant positive effect on animal performance when used for silage but Darby et al [10] observed no hybrid differences for forage, silage and stover yield.

Apart from the varying genetic background and harvest stage, differences between corn varieties are also affected by daily and seasonal temperature levels [8]. Corn in the tropics is fast growing but light is a major constraint to maximize the corn yield compared to the long summer days in the temperate area. Currently, most corn silage researches are in the temperate climate with limited findings in tropical countries especially Malaysia. As corn is primarily planted for food here and imported for feed, research must be focused on corn grown specifically for silage with the aim of providing alternative feed resources for the struggling beef industry. Hence, the study was done with the objectives of determining the optimum harvest stage of four corn varieties for tropical silage production in Malaysia based on the growth performance, yield, nutritive and feeding values as well as the financial feasibility for commercial production.

## MATERIALS AND METHODS

A field experiment was conducted at the farm of Department of Crop Science, Faculty of Agriculture, Universiti Putra Malaysia in Selangor, Malaysia (3° 2′ N, 101° 42′ E, 31 m above sea level) using a total area of 85.4 m×34 m. The average temperature during the planting period was 27°C with 78% relative humidity and 7.9 mm average daily rainfall. The project was carried out using split-plot design with four replications, harvest stage was the main plot while corn variety was the sub-plot. The four harvest stages selected were silking, milk, dough and dent stages. The corn varieties chosen comprised Sweet Corn hybrid 926 and three grain corn varieties; Suwan, breeding test lin (BTL) 1 and BTL 2. Both Sweet Corn and Suwan are commercial varieties while the other two were test lines from the faculty. Sweet Corn is the most commonly used corn variety in Malaysia specifically for human consumption while Suwan is a common type of grain corn.

The area was ploughed mechanically using disc plough to overturn the soil and samples were then collected for soil pH analysis. Liming was done at a rate of two tonnes/ha as the pH was lower than five. Further cultivation was done to break up the large soil particles using disc harrow followed by rotovation for suitable soil particle size for planting. Corn seeds were planted manually at a planting density of 66,667 plants per hectare. The planting distance was 75 cm between rows and 20 cm between plants. To ensure optimum growth of the plants, regular maintenance was carried out during the plant growth. Thinning of excess plants was done two weeks after planting while fertilizer was applied three times at a total rate of 140 kg N/ha, 100 kg P/ha and 120 kg/K ha. NPK green fertilizer (15N:15P_2_O_5_:15K_2_O), NPK blue fertilizer (12N: 12P_2_O_5_:17K_2_O) and urea fertilizer were applied several times within the planting period. Plastic mulching (Silvershine, Selangor, Malaysia) was used to inhibit weed growth but manual weed removal was also done frequently. The plot had a water sprinkler system as the source of irrigation with regular watering depending on the daily rainfall.

In each plot, two middle rows of two metre length were selected to avoid border effects from neighbouring plots. Each plant sample was harvested manually using sickle for precise harvesting with the stem cut 20 cm above the ground level. The following data were taken at sampling time using standard ruler and weighing machine: plant height, number of leaves, leaf, stem, cob, tassel, and whole plant fresh weight. The samples were then cut and dried by putting it into paper bags and placed in an oven at 60°C until constant weight [11]. The dry weight of the whole plant, leaves, stem, cob and tassel were then recorded. The DM content was calculated by dividing the dry weight with the fresh weight and converted into percentage. The dried samples were then ground using Retsch SM 100 cutting mill, with a screen of less than two mm particle size. The samples were then kept in pillboxes and stored in dry room until further analysis.

To measure the sample nutritive and feeding values, FOSS DS2500 Near Infrared Spectroscopy (NIRS) was used. Additional calibration was done using Mosaic Solo and WINISI 4 software using the samples analysed based on standard wet chemistry laboratory procedure. The crude protein (CP) composition was determined based on the method by AOAC [12] using Lachat Instrument 8000 series auto analyser machine while the neutral detergent fibre (NDF) and acid detergent fibre (ADF) were determined using FOSS FiberCap 2023 System (FOSS Analytical AB, Höganäs, Sweden) [13]. The lignin determination also used the method by ISO [13] using FOSS Fibertec M6 1020/1021 system (FOSS, Hilleroed, Denmark). The energy values were also measured using the formulae below based on the publication by NRC [14].

Digestible energy,DE (MJ/kg)=[0.04409×total digestible nutrient,TDN (%)]×4.184

Metabolizable energy,ME (MJ/kg)=[DE×0.82]×4.184

A detailed capital budgeting was done using selected harvest stage for each variety based on the prior field experiment. Due to lack of existing local business that can be used as reference, further data were obtained through several sources, either primary; questionnaire, interviews with the farmers, suppliers and contractors or secondary; publication from government agencies and research papers. All the data used was reviewed based on the prices in the state of Selangor, Malaysia for the period between January and May 2017. Assumptions about the project that are technically feasible were developed based on the field experiment with adjustments made for larger scale project including a 10% loss in yield adjustment. The capital budgeting method was done by creating a discounted cash flow based on the pooled data at 10% discount rate followed by financial appraisal that compare the net present value (NPV), internal rate of return (IRR), payback period, benefit cost ratio (BCR), profitability index (PI) and return of investment (ROI) [15].

### Statistical analysis

The data were analysed using analysis of variance (ANOVA) using SAS version 9.4 based on the split-plot design with two errors in the source of variation. Any significant differences between the treatment means were then compared using least significance difference (LSD) test of p value <0.05 and presented in mean. The mathematical model assumption used in ANOVA was:

$${\text{Y}}_{ijk}=\mu +{\text{B}}_{k}+{\text{H}}_{i}+{\text{S}}_{ik}+{\text{V}}_{j}+{\left(\text{HV}\right)}_{ij}+{\text{E}}_{ijk}$$

Where Y*_ijk_* is the dependent variable (growth performance; yield; nutritive value; feeding value etc.), *μ* is the mean, B*_k_* is the *k*th block effect, H*_i_* is the *i*th harvest stage (silking; milk; dough; dent) effect, S*_ik_* is the whole plot error, V*_j_* is the *j*th variety (Sweet Corn; Suwan; BTL 1; BTL 2) effect, (HV)*_ij_* is the effect of the *i*th harvest stage in *j*th variety and E*_ijk_* is the sub-plot error.

## RESULTS

Table 1 shows the growth parameters of the corn varieties at different harvest stage. There were significant differences in the leaf to stem (LS) ratio, DM content and cob to whole plant (CWP) ratio among the harvest stages. Significant differences were also observed among the different varieties in term of the plant height, leaf number, DM content and CWP ratio. Only the CWP ratio showed a significant interaction between the harvest stage and the variety. Generally, the plant height and the leaf number were constant across the harvest stages, but the LS ratio decreased while the DM content and CWP ratio increased. BTL 2 had the highest plant height and the highest leaf number while Sweet Corn had the least. In general, BTL 2 and BTL 1 had significantly higher DM content than Suwan and Sweet Corn.

Table 2 shows the yield parameters of the corn varieties at different harvest stage. There were significant differences in the total and cob DM yield among the harvest stages. Significant differences were also observed among the different varieties in term of the leaf, stem, tassel and total DM yield. Significant interactions between the harvest stage and the variety were detected only for the cob and total DM yield. Compared to the constant leaf, stem and tassel DM yield, the cob and total DM yield increased significantly across the harvest stages. While among the varieties, Sweet Corn generally had the lowest leaf, stem, tassel and total DM yield but the cob was not significantly different with BTL 1.

Table 3 shows the nutritive values of the corn varieties at different harvest stage. There were significant differences in the CP, NDF, ADF, and hemicellulose among the harvest stages. Significant differences were also observed among the different varieties in term of the CP and lignin. Significant interactions between the harvest stage and the variety were detected in the ADF and hemicellulose. As the harvest stage progressed, the CP and ADF decreased significantly while the NDF and hemicellulose increased but lignin remained constant. Compared to the grain corn varieties, the Sweet Corn generally had higher amount of CP with lower lignin content.

Table 4 shows the feeding values of the corn varieties at different harvest stage. There were significant effects of harvest stage but not for variety while the interactions between harvest stage and variety were all significant. There were significant differences in the digestible DM (DDM), TDN, DE, ME, net energy for maintenance (NEm) and net energy for gain (NEg) among the harvest stages. Generally, all the feeding value parameters had increased significantly across the harvest stage. Early in the silking stage, BTL 2 had the highest digestibility and energy content but later in the dent stage, the values were the lowest.

Table 5 shows the financial analysis result of the different corn varieties at selected harvest stage. The harvest stage selection was based on the highest yield with optimum quality. Sweet Corn production failed to yield any positive appraisal while all the grain corn varieties were profitable at a flat RM0.25/kg (0.06 USD/kg) selling price. In general, Suwan was the most viable as indicated by the highest positive NPV, BCR, ROI, PI, and shortest payback period.

## DISCUSSION

### Growth performance

Most of the varieties showed no significant difference among the harvest stages due to the corn plant finishing the vegetative growth as early as silking even with increasing DM yield in the later stages. The result was in contrast with Shehzad et al [16] who found significantly taller plant in a later harvest. The different results were possibly due to different plant growth stage at the selected harvest time. Sweet Corn had the shortest stem due to the variety characteristic that emphasizes the cob growth rather than stover development. As expected, the grain corn varieties had higher plant height to complement the higher leaf number, broader leaves and larger stem diameter with BTL 2 being the tallest variety.

In contrast with Sweet Corn, the taller grain varieties had more leaves which an important criterion for high yield silage corn plant. The leaf number was not significantly different among the harvest stages because of the vegetative growth completion during silking in tandem with the constant plant height. However, the leaf DM content can change significantly at a later maturity stage depending on the variety. As the DM increases, plant physical loss is more likely to happen due to weathering (wind and rainfall) and the leaf fraction sustains the most rapid and greatest losses [17].

In general, the LS ratio decreased significantly between silking and dough stage. As the plant becomes more mature or taller, the stem portion becomes greater while the leaves losses also increase due to maturity. This reduces the LS ratio with increasing plant maturity. It is better to harvest at higher LS ratio because the lower portion of the corn plant particularly the stem contains more fibre and lignin that would be less digestible. In general, early harvest can give better nutritive value but compromise must be given as the cob can heavily influence the total quality and it develops later than the leaf and stem. On average, the low yielding Sweet Corn has a similar ratio with the grain varieties because the plant short stature was balanced with smaller leaves compared to the grain corn varieties taller attribute with broader leaves. However, the Sweet Corn LS ratio decreased significantly at the end probably due to early stem maturity as it was developed to shift the plant metabolism towards the kernel production quickly.

The CWP ratio is an indicator of the quality of the whole plant yield as the cob specifically the kernel contains significant amount of CP with high amount of soluble carbohydrates and less fibre portion than the vegetative part. A higher CWP ratio is an important factor for forage nutritive value due to high digestibility of the grain. The cob kernel is abundant with starch as well as protein allowing the high cob content to compensate for the low general yield in term of quality rather than quantity [18]. In the experiment, Sweet Corn CWP ratio was the highest in all harvest stages even though it has the lowest yield because of the variety trait that focused on the cob development rather than whole plant biomass. While the different trends among the grain varieties show that similar corn type does not guarantee a similar plant development rate specifically in term of the cob production. Overall, the CWP ratio was the lowest early in the silking stage as the kernel was not yet developed thus having a very low cob DM yield. The CWP ratio then increased significantly as the harvest stage progressed similar to research by Opsi et al [11] that showed the contribution of grain to the whole crop increased with increasing maturity. Apart from that, BTL 2 ratio experienced a sudden significant decrease at the last dent stage due to the cob rapid DM decrease showing that the variety had matured faster.

At silking, the plant cob was not yet developed while the stem and leaves were still turgid causing the plant whole DM content to be low. The development of cob at the milk stage caused the plant moisture content to increase but the leaves, stem and tassel dried up faster significantly reducing the moisture content. As the plant reached the dough stage, the kernel starts to dry out thus further increasing the DM content. The plant DM continues to rise with advancing maturity and the increase can be significantly observed in all of the plant’s structures [19]. According to Keady et al [5], corn silage of 30% DM content can give the best animals growth rate while Lee et al [8] suggested harvest at near 35% DM content just before black layer formation to produce silage with optimum quality and yield. Compared to this research, the dough and dent stages gave the best DM content as it was the closest to the values suggested, though the optimum stage varies according to the varieties. The Sweet Corn DM content peaked early at the dough stage while Suwan at a latter dent stage. Both BTL 1 and 2 showed an early maturing characteristic as the DM content was high as early as the milk stage.

### Yield

At all stages, there was no significant difference in the leaf DM yield because the plant had completed the vegetative growth as early as silking. Therefore, no more leaves are developed in the later stages and this condition allows flexibility of harvest. Sweet Corn had significantly lower leaf DM yield because it was modified to emphasise on cob production for human consumption rather than for large biomass production. While Suwan, BTL 2 and BTL 1 can considered to be leafy varieties that had been developed to have high biomass with more leaves above the ears that can contribute to improved digestibility and better animal performance. Apart from the leafy trait, the stem was also much bigger and taller compared to Sweet Corn variety.

Like leaf DM yield, the stem DM yield also showed no significant difference among the harvest stages due to vegetative growth completion early in the silking stage without further stem elongation. This agrees with research by Darby et al [10] that found the maximum stover yield was reached at the time when the reproductive development initiates. Sweet Corn had the lowest stem DM yield among the varieties as expected, because of the distinctive characteristic of early maturing with short stem as the plant focus on cob development.

Generally, there was no significant difference in tassel DM yield among all growth stages because the plant had completed the tassel growth at silking stage. Although pollen was released for reproductive function, the amount was insignificant towards the tassel DM yield. The average tassel DM yield among the varieties had no significant difference except for Sweet Corn as the tassel was smaller, proportional to the plant small biomass.

Apart from high DM yield and optimum moisture content, high cob DM yield is a main factor contributing to high quality silage material. Sweet Corn was expected to give higher cob DM yield early on but instead, grain corn variety BTL 2 gave the highest cob DM yield starting from silking until the dent stage. However, the cob DM yield was similar across all varieties during milk and dent stages as the kernel was fully formed concurrently during the milk stage and later dries out in the dent stage. All the grain corn varieties showed an increase in cob DM yield across the harvest stages in agreement with research by Kim et al [20] that found large differences in ear percentage between silage corn hybrids at different harvest stage. Notably BTL 2 cob dried up faster than the other varieties at the dent stage thus reducing the cob DM yield significantly.

Similar to a study by Hetta et al [21], a quadratic total DM yield response to increasing maturity was observed within all tested varieties. The best harvest stage for each variety can be determined using the stage at which the plant DM yield was the highest. Based on this, Sweet Corn can be harvested as early as the milk stage while Suwan should be harvested only when it reaches the dent stage. Both BTL 2 and BTL 1 can be harvested either at dough or dent stages as there was no significant increase in the total DM yield. According to Pordesimo et al [17], the DM yield peaked when harvested at the time of grain physiological maturity, but it is not true for certain variety such as Sweet Corn as it was developed to be early maturing thus giving no increase in the total DM yield as early as the milk stage. In contrast, the grain corn varieties matured later at the dent stage as the total DM yield increased with crop maturity like research by Shehzad et al [16]. Compared to the commercial Suwan variety, both BTL 2 and BTL 1 showed that they are early maturing as the highest total DM yield was achieved earlier in the dough stage thus allowing flexibility in the harvest time.

### Nutritive values

During corn early growth stage, certain nutrient components especially CP is at the highest [22]. As the plant matures towards the dent stage, the kernel is filled with starch that effectively reduced the CP content. This combined with the dilution effect created by increasing grain portion, the CP content decreases more significantly as the corn plant matures [23]. In this research, the reduction was only significant in Suwan while the other grain varieties remained constant possibly due to the differences being too small to be significant or the error too high. As the Sweet Corn ear was well-developed, the CP content was high as expected due to the CP content concentrated in the ear portion of the corn plant. Even though Suwan gave similar mean CP content with Sweet Corn, the value was not significantly different from BTL 1 and BTL 2.

As the plant matures, the higher starch content with an increasing cob portion will effectively reduce the fibre content via dilution effect [23]. This reduction was observed in average, between silking and milk stages. While, the similarity between the latter harvest stages was in contrast with research by Zaralis et al [24] that observed a significantly lower NDF at the dent stage. Later increase recorded in the NDF content was similar to research by Estrada-Flores et al [19] that observed significant effects in leaves, stems, husk, rachis and whole plant. Even though there was a significant difference in term of the biomass production among the varieties, the average NDF content was similar across all varieties.

Hemicellulose is a structural carbohydrate that can be found in the plant cell wall and considered to be middle digestible fraction of corn plant. The cob and the husk had the highest amount of hemicellulose with the lowest amount of lignin, ash and mineral concentration [25]. As the cob developed rapidly at the silking stage, hemicellulose increased simultaneously with the cob development. This is evident by the hemicellulose increment for all varieties except for BTL 2. Sweet Corn recorded the highest amount of hemicellulose due to the variety genetic tendency to focus more on cob development. While Suwan and BTL 1 hemicellulose increased significantly early at milk stage proportional with the cob DM yield dramatic increase.

In contrast with the hemicellulose content increment, all the varieties showed decreasing ADF except for BTL 2 which has similar value across all harvest stages. The decline in the fibre concentration with increasing maturity was possibly due to the dilution effect caused by increasing grain content as the plant matures [22]. As lower ADF indicates higher digestibility, harvest is recommended at either dough or dent stage for both Sweet Corn and BTL 1 while Suwan and BTL 2 can be harvested earlier due to early ADF reduction. According to Chahine et al [26], normal ADF for corn silage is between 20% to 33% and based on this, BTL 2 exceeded that level in all harvest stages indicating lower overall quality in term of digestibility. The high ADF was probably due to significant ADF increment observed in the husk portion [19].

According to Darby et al [10], plant lignification process occurs as harvest dates progressed through the growing season. The lignification process is a result of the lignin deposition within the maturing cell wall in specific structural conformations [17]. The significant increase was observed in the stem and husk particularly [19]. But no significant difference was observed in lignin content among all harvest stages possibly due to early harvest time as lignification is prominent in the late dent until the black layer stage rather than the experiment dent stage [17]. As a later harvest did not increase the undesirable indigestible lignin, the harvest can be done at any harvest stage. On average, Sweet Corn had the lowest lignin content among the varieties due to shorter stem with larger cob portion that lower the lignin deposition in maturing stem cell wall.

### Feeding values

The general increases in DDM and TDN across the harvest stages provide more available digestible nutrients for the animal that can increase the growth potential significantly. DE was higher as it is the energy that was directly available when the feed was digested by the animals while ME resulted after the loss of energy in the urine. The experiment average ME value was less than a local research by Khaing et al [27] most likely due to varying fibre portion resulting from different corn variety and harvest time. However, compared to the research by Rahman et al [3], the ME value was still higher than conventional Napier grass, a popular feed among the farmers in Malaysia. Net energy (NE) is usually used with feed intake values to predict the animals gain from a diet. NEm and NEg are two NE components used to formulate diets specifically for growing and finishing cattle. As the results showed, the NEg was always lower than NEm because the feed energy was used more efficiently for maintenance than gain by the animals. Negative relationship can be seen between ADF and the feeding values, but the changing rate was different depending on the varieties genetic background. Though, a constant ADF across the harvest stages was observed in BTL 2 and had resulted in constant feeding value as well. Being one of the highly indigestible part of plant, the ADF increase had reduced the plant digestibility significantly and making it less efficient feed for the animal even with high feeding rate. The experiment digestibility and energy values shows flexibility for harvest in each variety, though the final selection must be paired with the yield and quality for optimum cattle performance.

### Financial analysis

The study showed that Sweet Corn was not financially viable for silage making purpose as the yield was too low and disproportionate with the input used. Even though it is the most popular variety in Malaysia, the variety advanced cob development trait was overshadowed by the plant relatively small biomass. Instead of planting for animal feed, Sweet Corn is better suited for human consumption as the cob is valued highly as food while the stover can be sold as by-product. Apart from positive cumulative cashflow, all the grain corn varieties also generated positive NPV and IRR values when considering the time value of money. Basically, the projects were accepted when the NPV value is greater than or equal to zero and the IRR is greater than the investor required rate of return (cost of capital), as going against this will cause loss and making it a senseless investment. Compared to ten years of production, the cost of investment was recovered early within a period of between two to less than four years. The short payback period indicated that the business models had low risk and good liquidity. Suwan had the highest PI among the financially viable grain corn varieties and the value indicated that the project was the most profitable as investor can expect RM2.86 (0.66 USD) return for every Ringgit invested. In addition, the ROI indicates a healthy 52.1% return on investment compared to the cost incurred. Compared to Iran, one of the world biggest market for grain, Suwan production BCR was not far off than their silage corn production [28]. The difference can be attributed to Iran’s better machinery usage and larger economies of scale. However, when compared to the world major corn producing countries such as Indonesia [29] and India [30], the BCR was much lower. The possible reasons were due to the absence of costly irrigation system, cheaper labour cost and lower amount of input due to larger economies of scale for the major corn producing countries.

In conclusion, generally Sweet Corn had the lowest yield but was slightly superior in term of the nutritive quality mostly due to consistently high CP content. On the other hand, the grain corn varieties (Suwan, BTL 1 and BTL 2) had much higher yield while the quality varies significantly. In general, early at silking stage, the CP, NDF, and ADF contents were at the highest but the values decreased over time in contrast with the DM yield, DDM, TDN, and energy content which increased over time. As the yield and quality rate of change were different depending on the varieties, the optimum harvest stage differs. For Sweet Corn, the optimum harvest stage was early in the dough stage, Suwan at the later dent stage while both BTL 1 and BTL 2 varieties had the flexibility to be harvested either at the dough or dent stage. Among the high yielding grain corn varieties, Suwan had the highest potential for commercial silage production as the financial analysis showed that it was the most profitable due to the highest financial appraisal values with the shortest payback period. While Sweet Corn was not suitable for silage at all as the yield was too low to even return the initial investment of planting.

## Figures and Tables

**Table 1:** Growth parameters of the different corn varieties at different harvest stage

Items	Plant height (cm)	Leaf number	LS ratio	CWP ratio	DM content (%)
ANOVA					
Harvest stage (H)	ns	ns	*	**	*
Variety (V)	**	**	ns	*	**
H×V	ns	ns	ns	**	ns
Sweet Corn					
Silking	154.7^a^	8.5^a^	0.71^a^	0.3^c^	16.4^c^
Milk	161.4^a^	8.1^a^	0.68^a^	0.7^a^	21.6^b^
Dough	158.4^a^	8.3^a^	0.66^ab^	0.5^b^	27.2^a^
Dent	150.8^a^	8.0^a^	0.54^b^	0.8^a^	23.7^ab^
Mean	156.3^C^	8.2^C^	0.65^A^	0.6^A^	22.3^B^
Suwan					
Silking	197.5^a^	11.6^ab^	0.63^a^	0.2^b^	19.4^c^
Milk	208.7^a^	11.7^ab^	0.64^a^	0.5^ab^	22.7^bc^
Dough	206.0^a^	11.2^b^	0.55^a^	0.6^a^	27.5^ab^
Dent	205.1^a^	11.8^a^	0.58^a^	0.6^a^	28.2^a^
Mean	204.3^B^	11.6^B^	0.60^A^	0.5^AB^	24.4^B^
BTL 2					
Silking	217.9^a^	12.1^a^	0.69^a^	0.4^ab^	22.8^b^
Milk	214.6^a^	11.6^a^	0.56^b^	0.5^ab^	31.2^ab^
Dough	216.3^a^	12.0^a^	0.55^b^	0.6^a^	31.1^ab^
Dent	221.5^a^	12.0^a^	0.61^ab^	0.4^b^	32.1^a^
Mean	217.6^A^	11.9^A^	0.60^A^	0.5^AB^	29.3^A^
BTL 1					
Silking	208.0^ab^	11.6^ab^	0.67^a^	0.3^b^	18.6^b^
Milk	204.9^ab^	11.8^a^	0.64^a^	0.4^ab^	28.4^ab^
Dough	194.6^b^	11.0^b^	0.48^a^	0.4^ab^	36.2^a^
Dent	210.0^a^	11.9^a^	0.60^a^	0.5^a^	31.1^ab^
Mean	204.4^B^	11.6^B^	0.60^A^	0.4^B^	28.6^A^

LS, leaf to stem; CWP, cob to whole plant; DM, dry matter; ANOVA, analysis of variance; ns, no significant difference; BTL, breeding test line.

* Significant at p<0.05;

** significant at p<0.01.

Harvest means within each variety having similar small letters are not significantly different, variety means with similar capital letters are not significantly different.

**Table 2:** Yield parameters of the different corn varieties at different harvest stage

Items	Leaf DM yield	Stem DM yield	Tassel DM yield	Cob DM yield	Total DM yield
ANOVA					
Harvest stage (H)	ns	ns	ns	**	**
Variety (V)	**	**	**	ns	**
H×V	ns	ns	ns	*	*
Sweet Corn					
Silking	2.42^a^	2.50^a^	0.25^c^	2.4^c^	8.5^b^
Milk	3.24^a^	4.95^a^	0.44^a^	9.7^ab^	13.2^a^
Dough	2.55^a^	3.92^a^	0.31^bc^	7.3^b^	15.3^a^
Dent	2.28^a^	4.26^a^	0.38^ab^	10.6^a^	13.7^a^
Mean	2.62^B^	3.91^B^	0.34^B^	7.5^B^	12.7^B^
Suwan					
Silking	3.41^a^	5.48^a^	0.49^a^	2.9^c^	12.7^c^
Milk	4.11^a^	6.45^a^	0.44^a^	8.9^bc^	19.0^b^
Dough	3.97^a^	7.31^a^	0.39^a^	12.2^ab^	20.2^b^
Dent	4.78^a^	8.70^a^	0.51^a^	17.7^a^	28.6^a^
Mean	4.07^A^	6.98^A^	0.46^A^	10.4^A^	20.1^A^
BTL 2					
Silking	5.70^a^	8.15^a^	0.72^a^	7.4^b^	17.4^c^
Milk	4.13^a^	7.47^a^	0.42^b^	10.2^b^	20.3^bc^
Dough	4.95^a^	9.06^a^	0.55^ab^	15.7^a^	25.3^ab^
Dent	4.37^a^	7.31^a^	0.46^ab^	10.3^b^	27.4^a^
Mean	4.79^A^	7.99^A^	0.53^A^	10.9^A^	22.6^A^
BTL 1					
Silking	3.83^a^	5.97^a^	0.47^a^	3.7^b^	12.8^b^
Milk	4.71^a^	7.44^a^	0.56^a^	7.6^ab^	18.8^b^
Dough	3.67^a^	7.81^a^	0.56^a^	12.9^a^	27.9^a^
Dent	4.07^a^	6.76^a^	0.50^a^	13.4^a^	28.1^a^
Mean	4.07^A^	6.99^A^	0.52^A^	9.4^AB^	21.9^A^

DM, dry matter (All the means are presented in t/ha.); ANOVA, analysis of variance; ns, no significant difference; BTL, breeding test line.

* Significance at p<0.05;

** significance at p<0.01.

Harvest means within each variety having similar small letters are not significantly different, variety means with similar capital letters are not significantly different.

**Table 3:** Nutritive values of the different corn varieties at different harvest stage

Items	CP1)	NDF1)	Hemi-cellulose	ADF1)	Lignin
ANOVA					
Harvest stage (H)	*	**	**	**	ns
Variety (V)	**	ns	ns	ns	**
H×V	ns	ns	**	**	ns
Sweet Corn					
Silking	11.7^a^	66.2^a^	22.1^c^	44.1^a^	5.92^a^
Milk	11.7^a^	60.0^b^	24.5^c^	35.4^b^	6.35^a^
Dough	10.7^a^	62.9^ab^	32.2^b^	30.8^bc^	6.02^a^
Dent	11.7^a^	65.2^a^	38.7^a^	26.5^c^	5.49^a^
Mean	11.4^A^	63.6^A^	29.4^A^	34.2^A^	5.95^C^
Suwan					
Silking	11.8^a^	65.2^a^	21.8^b^	43.4^a^	7.08^a^
Milk	11.2^ab^	63.8^a^	31.0^a^	32.8^b^	7.45^a^
Dough	10.7^b^	64.5^a^	30.4^a^	34.1^b^	7.44^a^
Dent	9.6^c^	63.5^a^	32.2^a^	31.3^b^	7.66^a^
Mean	10.8^AB^	64.2^A^	28.8^AB^	35.4^A^	7.41^A^
BTL 2					
Silking	11.2^a^	64.6^a^	27.4^a^	37.2^a^	5.44^a^
Milk	10.7^a^	61.1^b^	24.9^a^	36.2^a^	6.72^a^
Dough	8.7^a^	62.3^ab^	27.4^a^	34.9^a^	6.91^a^
Dent	9.8^a^	64.0^ab^	26.5^a^	37.5^a^	7.16^a^
Mean	10.1^B^	63.0^A^	26.5^B^	36.5^A^	6.55^BC^
BTL 1					
Silking	11.1^a^	63.8^a^	20.6^b^	43.3^a^	6.60^a^
Milk	10.3^a^	65.3^a^	28.3^a^	37.1^b^	6.97^a^
Dough	9.89^a^	63.2^a^	29.4^a^	33.8^bc^	7.03^a^
Dent	9.69^a^	63.9^a^	33.4^a^	30.6^c^	7.14^a^
Mean	10.2^B^	64.1^A^	27.9^AB^	36.2^A^	6.93^AB^

ANOVA, analysis of variance; ns, no significant difference; BTL, breeding test line.

1) CP, crude protein; NDF, neutral detergent fibre; ADF, acid detergent fibre. All the means are presented in percentages.

* Significance at p<0.05;

** significance at p<0.01.

Harvest stage means within each variety having similar small letters are not significantly different, variety means with similar capital letters are not significantly different.

**Table 4:** Feeding values of the different corn varieties at different harvest stage

Items	DDM (%)	TDN (%)	DE (MJ/kg)	ME (MJ/kg)	NEm (MJ/kg)	NEg (MJ/kg)
ANOVA						
Harvest stage (H)	**	**	**	**	**	**
Variety (V)	ns	ns	ns	ns	ns	ns
H×V	**	**	**	**	**	**
Sweet Corn						
Silking	54.6^c^	56.7^c^	10.5^c^	8.58^c^	5.02^c^	2.64^c^
Milk	61.3^b^	61.7^b^	11.4^b^	9.33^b^	5.69^b^	3.31^b^
Dough	65.0^ab^	64.4^ab^	11.9^ab^	9.75^ab^	6.11^ab^	3.64^ab^
Dent	68.3^a^	66.8^a^	12.3^a^	10.13^a^	6.40^a^	3.93^a^
Suwan						
Silking	55.1^b^	57.1^b^	10.5^b^	8.66^b^	5.06^b^	2.72^b^
Milk	63.3^a^	63.2^a^	11.7^a^	9.58^a^	5.94^a^	3.47^a^
Dough	62.4^a^	62.5^a^	11.5^a^	9.46^a^	5.82^a^	3.39^a^
Dent	64.5^a^	64.1^a^	11.8^a^	9.71^a^	6.02^a^	3.60^a^
BTL 2						
Silking	59.9^a^	60.7^a^	11.2^a^	9.16^a^	5.56^a^	3.18^a^
Milk	60.7^a^	61.2^a^	11.3^a^	9.29^a^	5.65^a^	3.22^a^
Dough	61.7^a^	62.0^a^	11.5^a^	9.37^a^	5.77^a^	3.35^a^
Dent	59.7^a^	60.5^a^	11.2^a^	9.16^a^	5.56^a^	3.14^a^
BTL 1						
Silking	55.2^c^	57.2^c^	10.5^c^	8.66^c^	5.10^c^	2.72^c^
Milk	60.1^b^	60.8^b^	11.2^b^	9.20^b^	5.61^bc^	3.18^bc^
Dough	62.6^ab^	62.6^ab^	11.5^ab^	9.46^ab^	5.86^ab^	3.43^ab^
Dent	65.1^a^	64.5^a^	11.9^a^	9.75^a^	6.11^a^	3.64^a^

DDM, digestible dry matter; TDN, total digestible nutrient; DE, digestible energy; ME, metabolizable energy; NEm, net energy for maintenance; NEg, net energy for gain; ANOVA, analysis of variance; ns, no significant difference; BTL, breeding test line.

* p<0.05;

** p<0.01.

Means with different letters vertically within each variety are significantly different.

**Table 5:** Financial appraisal of the different corn varieties at the selected harvest stage

Variety	NPV (RM)	IRR (%)	PBP	BCR	ROI (%)	PI
Sweet Corn	−271,356.21	−29.75	-	0.84	−15.6	−0.15
Suwan	439,999.15	40.04	2 yr. 7 mo.	1.52	52.1	2.86
BTL 2	189,654.79	24.12	3 yr. 10 mo.	1.28	28.3	1.80
BTL 1	266,726.19	29.23	3 yr. 4 mo.	1.36	35.6	2.13

NPV, net present value; IRR, internal rate of return; PBP, payback period; BCR, benefit cost ratio; ROI, return of investment; PI, profitability index; yr, years; mo, months; BTL, breeding test line.
